# Inhibition of the Notch1 Pathway Promotes the Effects of Nucleus Pulposus Cell-Derived Exosomes on the Differentiation of Mesenchymal Stem Cells into Nucleus Pulposus-Like Cells in Rats

**DOI:** 10.1155/2019/8404168

**Published:** 2019-05-06

**Authors:** Wei-ren Lan, Sai Pan, Hai-yin Li, Chao Sun, Xian Chang, Kang Lu, Chang-qing Jiang, Rui Zuo, Yue Zhou, Chang-qing Li

**Affiliations:** ^1^Department of Orthopedic, The Second Affiliated Hospital of Army Medical University (The Third Military Medical University), Chongqing, China; ^2^Department of Nephrology, General Hospital of PLA, Beijing, China

## Abstract

Stem cell therapies for intervertebral disc degeneration have been demonstrated as a promising strategy. Previous studies have shown that human nucleus pulposus cell- (NPC-) derived exosomes can induce the differentiation of mesenchymal stem cells (MSCs) into NP-like cells in vitro. However, the mechanism of MSC differentiation into NP-like cells with the induction of NPC exosomes is still unclear. Here, we verified the induction effects of NPC exosomes on the differentiation of MSCs into NP-like cells. In addition, the Notch1 pathway was downregulated in this process. Then, DAPT and soluble Jagged1 (SJAG) were applied to inhibit or enhance the expression of the Notch1 pathway, respectively, resulting in the upregulation or downregulation of collagen II, aggrecan, and Sox9 in MSCs. Knocking down of Notch1 protein facilitated the effects of NPC exosomes on the differentiation of MSCs into NP-like cells. NPC exosomes were more effective than an indirect coculture system in terms of the differentiation of MSCs into NP-like cells. Inhibition of NPC exosome secretion with Rab27a siRNA prevented the induction effects of an indirect coculture system on the differentiation of MSCs into NP-like cells. Transwell migration assays revealed that NPC exosomes could promote the migration of MSCs. Taken together, the Notch1 pathway was negatively associated with the differentiation of MSCs into NP-like cells with the treatments of NPC exosomes. Inhibition of the Notch1 pathway facilitates NPC exosome-induced differentiation of MSCs into NP-like cells in vitro. NPC exosomes play a key role in the differentiation of MSCs into NP-like cells in an indirect coculture system of NPCs and MSCs.

## 1. Introduction

Intervertebral disc degeneration (IDD) is one of the causes of low back pain [1]. Traditional treatments mainly focus on symptomatic relief. However, there are still some novel cell therapies for IDD, such as NPC transplantation [2] and MSC transplantation [3]. When IDD occurs, the proliferation activity and number of NPCs in intervertebral disc decrease, followed by the decrease of extracellular matrix synthesis [4]. Therefore, it is quite difficult to obtain sufficient autologous NPCs. MSC is one of the main cell sources of intervertebral disc tissue engineering because of its ability of multidirectional differentiation and self-renewal. Moreover, MSCs have the characteristics of easy access and low immunogenicity, which provide a possibility for stem cell transplantation in vivo. Many studies have shown that MSCs can differentiate into NP-like cells [5–7]. The key of stem cell therapy is to find an efficient way to induce MSC differentiation into NP-like cells.

Exosomes are extracellular vesicles of 30-100 nm in diameter secreted by cells [8]. It can deliver lots of bioactive substances, such as lipids, nucleic acids, and proteins to the recipient cells by membrane fusion, which can play the role of intercellular material exchange and information communication [9]. Numerous studies have shown that exosomes play an important role in tumor metastasis [10], immunomodulation [11], and repair of myocardial injury [12]. In addition, exosomes can induce the differentiation of stem cells. For example, exosomes derived from hematopoietic stem cells can induce hematopoietic differentiation of embryonic stem cells [13]. Our previous study has found that human NPC-derived exosomes can induce MSC differentiation into NP-like cells [14]. These studies indicate that using exosomes to induce the differentiation of MSCs into NPCs is a promising strategy. However, the specific mechanism of exosome-mediated differentiation of MSCs into NP-like cells and on how to regulate this differentiation process is still unknown.

It has been widely reported that the Notch signaling pathway is involved in the regulation of cell proliferation [15], differentiation [16], and apoptosis [17]. Activation of the Notch pathway with dihydroartemisinin can inhibit the differentiation of MSCs into chondrocytes [18]. Besides, under the use of oleanolic acid, the inhibition of the Notch pathway can promote the differentiation of MSC into osteoblasts [19]. There exist five Notch ligands (delta-like homologues 1, 3, and 4, Jagged1, and Jagged2) and four Notch receptors (Notch1-4) in mammals [20]. Among them, Notch1 is also involved in the process of proliferation and differentiation of various cells. For example, the activation of the Notch1/Jagged1 pathway inhibits the differentiation of epidermal stem cells into myofibroblasts [21]. Obviously, the Notch pathway is involved in the differentiation of MSCs under various conditions and has played an inhibitory role. Therefore, we speculate that the Notch pathway is involved in exosome-induced differentiation of MSCs into NPCs.

In this study, we explored the differential expression of the Notch pathway in exosome-induced differentiation of MSCs into NP-like cells and further explored its role by enhancing or inhibiting the Notch pathway. Furthermore, we investigated whether the inhibition of Notch1 could promote the exosome-induced differentiation of MSCs. Finally, we compared the inducing efficiency of NPC exosomes with an indirect coculture system on the differentiation of MSCs into NP-like cells and explored the role of NPC exosomes in an indirect coculture system by the inhibition of exosome secretion.

## 2. Materials and Methods

### 2.1. Cell Isolation and Culture

MSCs and NPCs were isolated from eight-week-old male Sprague-Dawley rats, which were brought from Animal Centers of the Second Affiliated Hospital of Army Medical University. All procedures were approved by the Ethics Committee of the Second Affiliated Hospital of Army Medical University. NPCs and MSCs were isolated using methods reported by Risbud et al. [22] and Chang et al. [23], respectively. Briefly, rats were sacrificed under the condition of anesthesia. Coccygeal vertebra, femurs, and tibias were removed under aseptic condition. The nucleus pulposus tissue was separated from coccygeal vertebra and cut into small pieces. After 6-hour digestion with 0.1% collagenase, the tissue suspension was centrifuged at 300 g for 5 min and then resuspended in Dulbecco's modified Eagle's medium (DMEM)-F12 medium with 10% fetal bovine serum and 100 U/ml penicillin-streptomycin. Then, the partially digested tissue was incubated in humidified atmosphere containing 5% CO_2_ at 37°C. After 1-week incubation, NPCs migrated from the partially digested tissue. Bone marrow was separated from femurs and tibias, soaked in DMEM-F12 medium with 10% fetal bovine serum and 100 U/ml penicillin-streptomycin, and then dispersed by 16-gauge needles. After that, cells were plated in culture flasks in humidified atmosphere containing 5% CO_2_ at 37°C. Nonadherent cells were removed by changing the medium after 48 h. When reaching 90% confluence, both NPCs and MSCs were washed twice with phosphate-buffered saline (PBS), then digested by 0.25% trypsin, and subcultured at 1 : 2. Culture medium was changed every 3 days.

### 2.2. Flow Cytometry Analysis of Rat MSCs

Passage 3 rat MSCs were digested by 0.25% trypsin, washed twice with PBS, and then resuspended in PBS as single cell suspension. The cells were incubated with fluorescence-coupled antibodies and isotype control antibodies, respectively (0.4 *μ*g antibody: 1 × 10^5^ cells). The antibodies involved are as follows: PE anti-mouse/rat CD29 antibody (BioLegend, USA, catalog number: 102207), FITC anti-rat CD90 antibody (BioLegend, USA, catalog number: 206105), FITC mouse anti-rat CD44H (BD Biosciences, USA, catalog number: 550974), CD34 antibody-FITC (Santa Cruz Biotechnology, USA, catalog number: sc-7324 FITC), CD45 antibody-FITC (Santa Cruz Biotechnology, USA, catalog number: sc-1178 FITC), PE Armenian hamster IgG isotype Ctrl antibody (BioLegend, USA, catalog number: 400907), and FITC mouse IgG1, *κ* isotype Ctrl antibody (BioLegend, USA, catalog number: 400107). After 30 min of incubation at 4°C in the dark, rat MSCs were washed twice with PBS and resuspended in 300 *μ*l PBS for analysis. Cell fluorescence was examined by a FACScan flow cytometer running CellQuest software.

### 2.3. Differentiation Potentials of Rat MSCs

To evaluate the differentiation potentials of rat MSCs, osteogenic, adipogenic, and chondrogenic differentiation of MSCs was performed according to the manufacturer's protocol.

#### 2.3.1. Osteogenic Differentiation

Passage 3 MSCs were plated in 6-well plates at 2 × 10^4^ cells/cm^2^ density with a complete culture medium. When reaching 80-90% confluence, the culture medium was changed with the osteogenic differentiation medium (Cyagen Biosciences Inc., catalog number: RASMX-90021) and then changed every 3 days. Each kit of the osteogenic differentiation complete medium contains 175 ml basal medium, 20 ml fetal bovine serum, 2 ml penicillin-streptomycin, 2 ml glutamine, 2 ml *β*-glycerophosphate, 400 *μ*l ascorbate, and 20 *μ*l dexamethasone. After 21 days of induction, MSCs were fixed with 4% paraformaldehyde and stained by alizarin red solution.

#### 2.3.2. Adipogenic Differentiation

Passage 3 MSCs were plated in 6-well plates at 2 × 10^4^ cells/cm^2^ density with a complete culture medium. When confluency reached 100%, adipogenic differentiation medium A (Cyagen Biosciences Inc., catalog number: RASMX-90031) was added for 3 days and then was replaced by adipogenic differentiation medium B (Cyagen Biosciences Inc., catalog number: RASMX-90031) for 24 h. Each kit of adipogenic differentiation medium A contains 175 ml basal medium, 20 ml fetal bovine serum, 2 ml penicillin-streptomycin, 2 ml glutamine, 400 *μ*l insulin, 200 *μ*l IBMX, 200 *μ*l dexamethasone, and 200 *μ*l rosiglitazone. Each kit of the adipogenic differentiation medium B contains 175 ml basal medium, 20 ml fetal bovine serum, 2 ml penicillin-streptomycin, 2 ml glutamine, and 400 *μ*l insulin. Three cycles later, the MSCs were fixed with 4% paraformaldehyde and stained by oil red O solution.

#### 2.3.3. Chondrogenic Differentiation

The pellet culture was performed to induce chondrogenic differentiation. Approximately 2.5 × 10^5^ cells were centrifuged as a pellet at 150 g for 5 min in a 15 ml polypropylene tube. The supernatant was discarded and changed with the chondrogenic differentiation medium (Cyagen Biosciences Inc., catalog number: RASMX-90041) carefully. Each kit of the chondrogenic differentiation complete medium contains 194 ml basal medium, 600 *μ*l ascorbate, 20 *μ*l dexamethasone, 2 ml ITS, 200 *μ*l sodium pyruvate, 200 *μ*l proline, and 2 ml TGF-*β*3. After 21 days, the pellet was fixed with 4% paraformaldehyde, embedded in paraffin wax, cut into thick sections, and stained with alcian blue solution.

### 2.4. Isolation of NPC Exosomes

NPC exosomes were isolated by differential centrifugation as previously described [24]. Briefly, NPCs were cultured in DMEM-F12 medium containing 10% fetal bovine serum depleted of exosomes by ultracentrifugation at 120,000 g for 90 min. After 48 h, the medium was collected and centrifuged at 300 g for 10 min at 4°C to eliminate cells and at 20,000 g for 30 min to remove apoptotic body and microvesicles. At each step, only 95% of the supernatant from the upper surface was collected. The remaining 5% of the supernatant and the pellets were discarded. Then, the supernatant was filtered with a 0.22 *μ*m membrane to remove the particles larger than 0.22 *μ*m in diameter. Exosomes were pelleted by ultracentrifugation at 120,000 ×g for 70 min at 4°C and then washed and resuspended in PBS. The exosomes were quantified by Bradford protein assay and stored at -80°C for further use.

### 2.5. Identification of NPC Exosomes

#### 2.5.1. Transmission Electron Microscopy

NPC exosomes were obtained through differential centrifugation and resuspended in PBS. 10 *μ*g exosome suspension was dropped onto electron microscopy copper grids and dried for 10 min. After that, the grids were stained with 1% phosphotungstic acid for 5 min and examined by using a transmission electron microscope (JEM-1400Plus, Japan) at 100 kV.

#### 2.5.2. Western Blot Analysis of Exosome Surface Markers

The expression of CD63, CD81, Tsg101, and calnexin in exosomes was detected by Western blot following the protocols.

### 2.6. Exosome Labeling and Fluorescence Microscopy

Exosomes and MSCs were labeled by fluorescence dye CM-Dil and CM-Dio, respectively, as previously described [25]. Briefly, 20 *μ*g exosomes in 100 *μ*l PBS was incubated with CM-Dil in the dark for 30 min, washed with PBS, ultracentrifuged at 120,000 g for 70 min to remove nonbinding dye, and then resuspended in PBS. MSCs were incubated with CM-Dio in the dark for 30 min and centrifuged at 300 g for 5 min to remove nonbinding dye. The CM-Dil labeled exosomes were incubated with CM-Dio-labeled MSCs at 37°C for 24 h. After the incubation, the fluorescence images were collected by using a fluorescence microscope and analyzed with the Leica Application Suite Advanced Fluorescence software.

### 2.7. Quantitative RT-PCR Analysis

Each step of qRT-PCR was accomplished according to the manufacturer's protocols. After treatments, MSCs and NPCs were washed twice with PBS and treated with the TRIzol reagent (Invitrogen, CA, USA) to extract RNA. PrimeScript RT reagent kit (Takara, Japan) was used for RNA reverse transcription to synthesize cDNA. SYBR Premix Ex Taq II Kit (Takara, Japan) was used for amplification to detect the relative mRNA expressions of target genes with the CFX96 Real-Time PCR System (Bio-Rad, Hercules, CA, USA). The reaction conditions were as follows: at 95°C for 30 s (1 cycle), at 95°C for 5 s, and at 60°C for 34 s (40 cycles). GAPDH was chosen as a reference gene to normalize the levels of the target genes. The primers for qRT-PCR were presented in Table 1. Relative mRNA expressions were calculated by a comparative threshold cycle (Ct) method using the formula 2^‐ΔΔCt^.

### 2.8. Western Blot Analysis

MSCs and exosomes were lysed with cell lysis buffer (Beyotime Biotechnology) containing 1% PMSF (Beyotime Biotechnology) and then centrifuged at 14,000 g and 4°C for 5 min to discard the cell debris. MSC lysates and exosome lysates were subjected to sodium dodecyl sulfate-polyacrylamide gel electrophoresis and transferred to 0.45 *μ*m polyvinylidene difluoride membranes (Millipore). The membranes were blocked by 5% nonfat milk in TBST, incubated with specific antibodies overnight at 4°C, and then washed with TBST three times. Besides, the membranes were incubated with horseradish peroxidase-labeled secondary antibody (dilution 1 : 4000) for 2 h and visualized using an enhanced chemiluminescence substrate (Bio-Rad) and ImageQuant LAS 4000. The relative expressions of target protein to GAPDH were calculated by using the ImageJ software. The antibodies involved are as follows: GAPDH (Beyotime, 1 : 1000 dilution), collagen II/aggrecan/CD63/CD81/Tsg101/calnexin/Jagged1/Notch1-4 (Santa Cruz, 1 : 200 dilution), Sox9 (Abcam, 1 : 1000 dilution), Hes1 (Zen Bioscience, 1 : 500 dilution), Hey1 (Affinity, 1 : 500 dilution), and Rab27a (Proteintech, 1 : 500 dilution).

### 2.9. DAPT and SJAG Treatments

Passage 3 MSCs were divided into four groups: the DAPT group, MSCs were treated with DAPT (Meilun Biotechnology, China, catalog number: MB5152) at a concentration of 20 *μ*M; the DAPT control group, MSCs were treated with dimethyl sulfoxide (DMSO); the SJAG group, MSCs were treated with SJAG (R&D Systems, USA, catalog number: 599-JG-100) at a concentration of 5 nM; and SJAG control group, MSCs were treated with PBS. The culture medium was changed every 3 days. After 14 days, MSCs were collected for analysis.

### 2.10. siRNA Transfection

Rats Notch1 and Rab27a siRNA were designed and synthesized by Shanghai Genechem Corporation (Shanghai, China). Notch1 siRNA sequence is 5′-GGGTGTATACTGTGAGATCA-3′. Rab27a siRNA sequence is 5′-GTGGGCATTGATTTCAGGGAA-3′. MSCs and NPCs were seeded in six-well plates and cultured until 20-30% confluence; then, MSCs were transfected with Notch1 siRNA or negative control, while NPCs were transfected with Rab27a siRNA or negative control. The medium was changed after 12 h. When reaching 80-90% confluence, the cells were passaged for further use.

### 2.11. Construction of an Indirect Coculture System

Here, 2 × 10^5^ NPCs were seeded in each well of six-well plates (Corning Inc., Corning, NY, USA). A transwell (Millicell Hanging Cell Culture Insert, PET 1 *μ*m, 6-well, Millipore, catalog number: MCRP06H48) covered with 2 × 10^5^ MSCs was inserted into the six-well plates. After 14 days of coculture, the MSCs were collected for further analyses.

### 2.12. Migration Assay of MSCs

For transwell migration assay, passage 3 MSCs were starved in the serum-free medium for 24 h. 1 × 10^5^ cells were then transferred to the upper chambers of the transwell system (Millicell Hanging Cell Culture Insert, PET 8 *μ*m, 24-well, Millipore, catalog number: MCEP24H48) with a 100 *μ*l serum-free medium. 500 *μ*l complete medium which contains NPC exosomes with the concentration of 25, 50, 75, and 100 *μ*g/ml was added to the lower chambers. After that, 500 *μ*l exosome-free complete medium was added as control. After 10-hour incubation, the MSCs covering on the upper surface of membranes were wiped off by using cotton swabs. Then, the membranes were fixed with 4% paraformaldehyde and stained with 0.1% crystal violet. A microscope was used to observe the migration of MSCs.

### 2.13. Statistical Analysis

Data were presented as mean ± standard deviation (SD). SPSS13.0 software (SPSS Inc., IL, USA) was used to conduct statistical analysis. The normality test of data was performed by the Shapiro-Wilk test. *P* value < 0.05 represented that data was normal distribution. Statistical analyses of normally distributed continuous variables were performed by Student's *t*-test or one-way analysis of variance (ANOVA) followed by Tukey's test. Statistical analyses of nonnormally distributed continuous variables were performed by Wilcoxon rank-sum test. *P* value < 0.05 was considered statistically significant.

## 3. Results

### 3.1. Phenotypic Characterization of Rat MSCs

**Figure 1:**
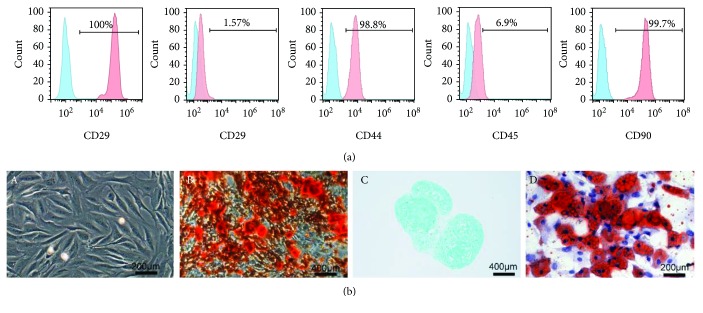
Phenotypic characterization of rat MSCs. (a) MSC immunophenotypic profiles were detected by using a flow cytometer. The red lines indicate the fluorescence intensity of cells stained with the corresponding antibodies, and the green lines represent isotype-matched negative control cells. (b) Passage 3 MSCs (A) were treated with the osteogenic differentiation medium for 21 days and then stained with alizarin red (B), alcian blue of the MSC pellet after chondrogenic induction for 21 days (C), and oil red O of MSCs after adipogenic induction for 12 days (D).

Rat MSCs were successfully isolated and subcultured to passage 3. MSC immunophenotypic profiles were detected by using a flow cytometer. MSCs were negative for CD34 and CD45 and positive for CD29, CD44, and CD90 (Figure 1(a)), which was similar to previously described MSCs [23]. Osteogenic, adipogenic, and chondrogenic differentiation of passage 3 rat MSCs was performed as manufacturer's protocols. For osteogenic differentiation, calcium nodules were visible after being stained by alizarin red solution (Figure 1(b)). After being treated with the adipogenic differentiation medium for 12 days, lipid droplets were observed in rat MSCs by using oil red O solution (Figure 1(b)). A pellet culture system was used for chondrogenic differentiation. Glycosaminoglycans were detected by alcian blue staining to prove the chondrogenic potential of rat MSCs (Figure 1(b)).

### 3.2. Identification of NPC Exosomes

**Figure 2:**
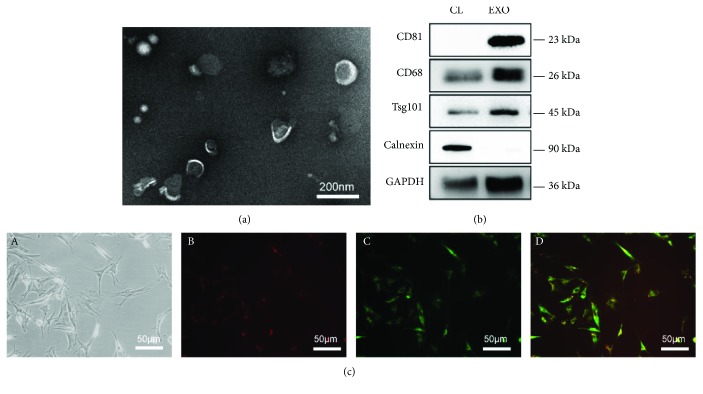
Identification of NPC exosomes and internalization of NPC exosomes by MSCs. (a) Characteristics of exosomes derived from NPCs under a transmission electron microscope. (b) Western blot analyses of exosomal protein markers CD63, CD81, and Tsg101 and negative protein calnexin. CL: NPC cell lysate; EXO: NPC exosomes. (c, A) Internalization of NPC exosomes by MSCs. NPC exosomes were stained with CM-Dil (B), and MSCs were stained with CM-Dio (C). Internalization was observed by a fluorescence microscope (D).

The transmission electron microscope showed that NPC exosomes were cup-shaped vesicles and <100 nm in size (Figure 2(a)). Besides, NPC exosomes were positive for exosomal marker protein CD63, CD81, and Tsg 101 and negative for endoplasmic reticulum-specific expression protein calnexin (Figure 2(b)). These results showed that the vesicles isolated from NPC culture supernatant could be identified as NPC exosomes.

### 3.3. Internalization of NPC Exosomes by MSCs

To detect the internalization of NPC exosomes by MSCs, NPC exosomes were labeled with CM-Dil (red) and incubated with CM-Dio (green)-labeled MSCs at 37°C for 24 h. As shown in Supplementary Figure 1, the red channel is stained exosomes but not precipitates of the dye. Red fluorescence spots were observed in green fluorescence-labeled MSCs under the fluorescence microscope (Figure 2(c)), which confirmed that NPC exosomes could be internalized by MSCs.

### 3.4. NPC Exosomes Can Induce MSC Differentiation into NP-Like Cells

**Figure 3:**
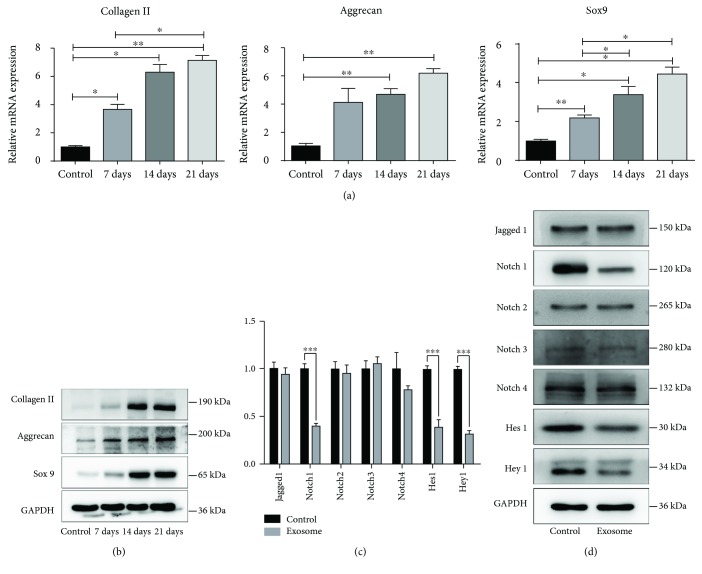
Expression of NPC markers and Notch pathway in NPC exosome-treated MSCs. (a) Gene expression of NPC markers (collagen II, Aggrecan, and Sox9) was significantly upregulated in exosome-treated MSCs in 7, 14, and 21 days. (b) Expression of related protein was normalized to GAPDH. (c) Expression of Notch pathway-related genes in exosome-treated MSCs was detected by qRT-PCR. (d) Western blot analysis of Notch pathway-related protein in exosome-treated MSCs. All data were showed as mean ± SD. *N* = 3. ^∗^ *P* < 0.05, ^∗∗^ *P* < 0.01, and ^∗∗∗^ *P* < 0.001.

To verify the induction effects of NPC exosomes on MSC differentiation into NP-like cells, Western blot and qRT-PCR were performed to detect the expression of collagen II, aggrecan, and Sox9 in MSCs after being incubated with NPC exosomes in 7, 14, and 21 days. After incubation, the expression of collagen II, aggrecan, and Sox9 increased significantly on mRNA and protein levels (Figures 3(a) and 3(b)). Also, the expression of NP markers CD24 and KRT19 increased on protein levels (Supplementary Figure 2). The induction effects of exosomes on the differentiation of MSCs into NPCs were mainly reflected in the upregulated expression of NP markers in MSCs, rather than morphology (Supplementary Figure 3).

### 3.5. Expression of Notch Signaling Pathway-Related Genes in NPC Exosome-Treated MSCs

To investigate whether the Notch signaling pathway was involved in the differentiation, we detected the expression of the Notch signaling pathway-related genes such as Jagged1, Notch1-4, hairy and enhancer of split-1 (Hes1), and Hes-related family BHLH transcription factor with YRPW motif 1 (Hey1) during differentiation. The results showed that Notch1, Hes1, and Hey1 decreased significantly in exosome-treated MSCs compared with control groups (Figures 3(c) and 3(d)). However, the expression of Jagged1, one of the Notch pathway ligands, had no significant difference between the exosome-treated MSC and control groups (Figures 3(c) and 3(d)).

### 3.6. Notch1 Pathway Was Negatively Associated with MSC Differentiation

**Figure 4:**
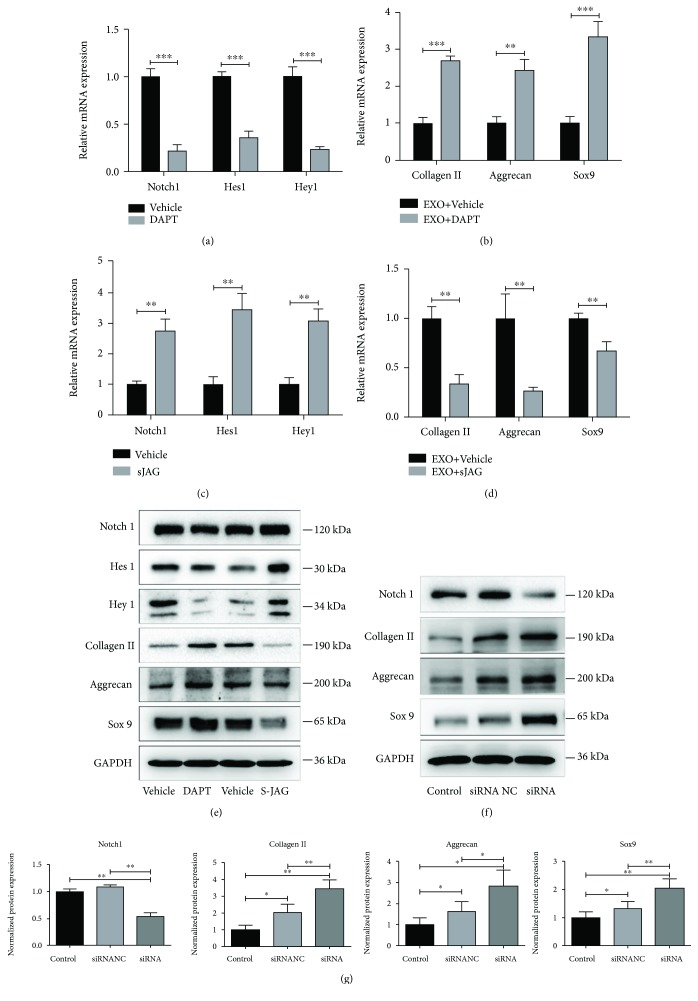
Notch1 pathway was negatively associated with exosome-induced MSC differentiation. (a) Gene expression of Notch1, Hes1, and Hey1 was significantly downregulated in DAPT-treated MSCs. Vehicle: DMSO. (b) DAPT enhanced the expression of collagen II, aggrecan, and Sox9 in exosome-treated MSCs. Vehicle: DMSO. (c) Gene expression of Notch1, Hes1, and Hey1 was significantly upregulated in SJAG-treated MSCs. Vehicle: PBS. (d) SJAG enhanced the expression of collagen II, aggrecan, and Sox9 in exosome-treated MSCs. Vehicle: PBS. (e) Western blot analysis of Notch1, Hes1, Hey1, collagen II, aggrecan, and Sox9 in MSCs treated under the same condition as (b) and (d). (f, g) Inhibition of Notch1 protein promoted the protein expression of collagen II, aggrecan, and Sox9 in exosome-treated MSCs. Control: MSCs treated with NPC exosomes (50 *μ*g/ml); siRNA NC: MSCs treated with NPC exosomes (50 *μ*g/ml) and Notch1 siRNA-negative control; siRNA: MSCs treated with NPC exosomes (50 *μ*g/ml) and Notch1 siRNA. All data were showed as mean ± SD. *N* = 3. ^∗^ *P* < 0.05, ^∗∗^ *P* < 0.01, and ^∗∗∗^ *P* < 0.001.

To investigate the roles of the Notch1 pathway in MSC differentiation, a *γ*-secretase inhibitor DAPT and a Notch ligand SJAG1 were used to pharmacologically inhibit or enhance the expression of the Notch1 pathway. As shown in Figure 4, DAPT significantly inhibited the expression of Notch1, Hes1, and Hey1 (Figure 4(a)), resulting in the upregulation of collagen II, aggrecan, and Sox9 expression in exosome-treated MSCs (Figure 4(b)). On the contrary, SJAG1 enhanced the expression of Notch1, Hes1, and Hey1 (Figure 4(c)), followed by the downregulation of collagen II, aggrecan, and Sox9 expression (Figure 4(d)). Protein expression of related genes is consistent with the mRNA expression (Figure 4(e)).

### 3.7. Knocking Down of Notch1 Protein Facilitates NPC Exosome-Induced Differentiation of MSCs into NP-Like Cells

MSCs were transfected with Notch1 siRNA to further demonstrate the effects of Notch1 on exosome-induced differentiation. The protein expression of Notch1 decreased significantly in MSCs transfected with Notch siRNA compared with MSCs transfected with siRNA-negative control or MSCs alone, followed by the upregulation of collagen II, aggrecan, and Sox9 expression (Figures 4(f) and 4(g)), but it had no significant difference between the siRNA-negative control and control groups.

### 3.8. NPC Exosomes Are More Effective Than an Indirect Coculture System on the Differentiation of MSCs into NP-Like Cells

**Figure 5:**
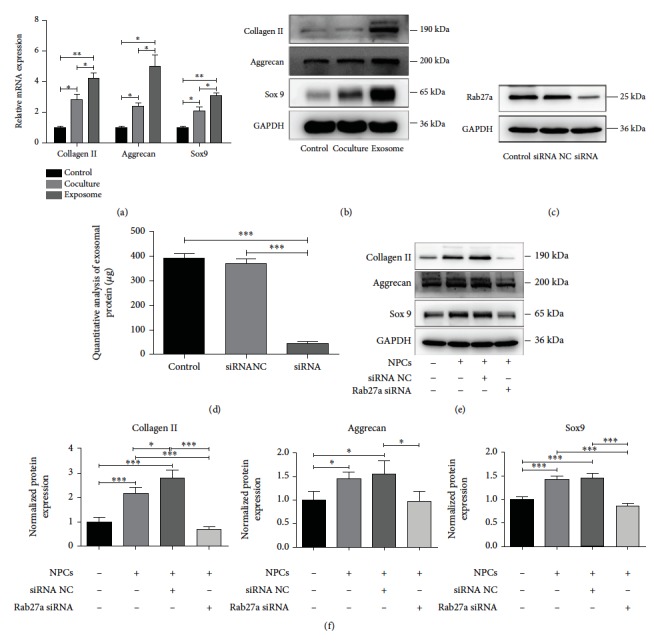
NPC exosomes played a key role in MSC differentiation into NP-like cells in an indirect coculture system. (a, b) Expression of collagen II, aggrecan, and Sox9 in the coculture group was higher than that in the control, but lower than the coculture group (*P* < 0.05). Control: MSCs cultured alone; coculture group: MSCs indirectly cocultured with NPCs; exosome group: MSCs treated with NPC exosomes (50 *μ*g/ml). (c) NPC Rab27a protein was knocked down via Rab27a siRNA transfection. (d) Exosomal proteins were extracted from the culture medium of the control group, siRNA NC group, and siRNA group and quantified with Bradford protein assay. Each group contain 1 × 10^6^ cells. Control: NPCs cultured alone; siRNA NC group: NPCs transfected with Rab27a siRNA-negative control; siRNA group: NPCs transfected with Rab27a siRNA. (e, f) Expression of collagen II, aggrecan, and Sox9 was analyzed by western blot analysis in MSCs, which were cocultured with NPCs transfected with Rab27a siRNA-negative control and Rab27a siRNA-negative control and Rab27a siRNA. All data were showed as mean ± SD. *N* = 3. ^∗^ *P* < 0.05, ^∗∗^ *P* < 0.01, and ^∗∗∗^ *P* < 0.001.

An indirect coculture system of MSCs and NPCs was performed to compare the induction effects of NPC exosomes on MSC differentiation. As shown in Figures 5(a) and 5(b), the expression of collagen II, aggrecan, and Sox9 in the coculture group was higher than that in the control group, but lower than the exosome group (*P* < 0.05).

### 3.9. Inhibition of NPC Exosome Secretion Prevents the Differentiation of MSCs into NP-Like Cells in an Indirect Coculture System

To investigate the roles of NPC exosomes in MSC differentiation in an indirect coculture system, Rab27a protein was knocked down via Rab27a siRNA to prevent NPC exosome secretion (Figure 5(c)). Knockdown of Rab27a attenuated exosome secretion (Figure 5(d)). Accordingly, the expression of collagen II, aggrecan, and Sox9 in MSCs cocultured with Rab27a siRNA-treated NPCs decreased significantly (Figures 5(e) and 5(f)). These results suggested that exosomes played a key role in MSC differentiation in an indirect coculture system.

### 3.10. NPC Exosomes Can Promote MSC Migration

**Figure 6:**
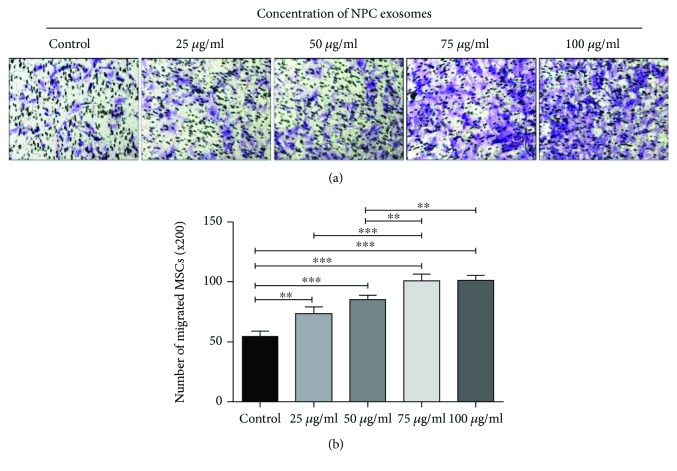
Transwell migration assay of MSCs. (a) Migration of MSCs treated with NPC exosomes at the concentration of 25 *μ*g/ml, 50 *μ*g/ml, 75 *μ*g/ml, and 100 *μ*g/ml was observed under the microscope (x200). (b) Numbers of migrated MSCs at the concentration of 25 *μ*g/ml, 50 *μ*g/ml, 75 *μ*g/ml, and 100 *μ*g/ml. All data were showed as mean ± SD. *N* = 3. ^∗^ *P* < 0.05, ^∗∗^ *P* < 0.01, and ^∗∗∗^ *P* < 0.001.

With the increasing concentration of exosomes, the amounts of migrated MSCs were increasing significantly until exosome concentration reached 75 *μ*g/ml (Figures 6(a) and 6(b)). These results indicated that NPC exosomes could promote MSC migration and 75 *μ*g/ml could be recommended as an optimal concentration for migration.

## 4. Discussion

The key to cell therapy is to find a cell source that can replenish degenerated NPCs. There have been many attempts to induce MSCs into NPCs, like treatment of growth factors TGF*β* and GDF6 [26], delivered by biomaterials [27], and construction of the coculture system [28]. Among them, the induction effect of the coculture system has been widely verified. However, its specific mechanism is not yet fully understood, which limits the further research. More and more studies have shown that exosomes play a mediating role in intercellular communication [29]. Here, we extracted and identified rat NPC exosomes (Figures 2(a) and 2(b)) and proved its effect on the differentiation of MSCs into NP-like cells in the monolayer culture system (Figures 3(a) and 3(b)), which is consistent with our previous study [14].

The role of the Notch pathway in inhibiting differentiation of various cells has been confirmed in several studies [30, 31]. In this study, we detected the expression of Notch pathway-related genes in NPC exosome-induced MSC differentiation and found that Notch1 and its downstream genes Hes1 and Hey1 decreased significantly. It suggested that the Notch1 pathway may be involved in exosome-induced MSC differentiation. To test this hypothesis, we used *γ*-secretase inhibitor DAPT and Notch pathway ligand SJAG to inhibit and activate the Notch1 pathway, respectively. With the induction of exosomes, the expression of Notch1, Hes1, and Hey1 decreased in DAPT-treated MSCs compared with the vehicle group, followed by increased expression of collagen II, aggrecan, and Sox9. The expression of Notch1, Hes1, and Hey1 increased in the SJAG-treated MSCs compared with the vehicle group, resulted in decreased expression of collagen II, aggrecan, and Sox9. These results indicated that the expression of the Notch1 pathway is negatively correlated with exosome-induced MSC differentiation. To further verify this hypothesis, siRNA transfection was performed to knock down Notch1 protein, followed by increased expression of collagen II, aggrecan, and Sox9. It showed that the inhibition of the Notch1 pathway could promote NPC exosome-induced differentiation of MSCs into NP-like cells.

Stem cell reserve in intervertebral disc is the basis for self-repair and regeneration of the intervertebral disc, like cartilage endplate stem cells [32] and annulus fibrosus stem cells [33]. Promoting the migration of stem cells into nucleus pulposus is a promising strategy for replenishment of NPCs. This experiment demonstrated that NPC exosomes could promote the migration of MSCs, and the migration rate of MSCs was concentration-dependent (Figures 6(a) and 6(b)). It provided a theoretical basis for further experiments of NPC exosomes in vivo. In addition, this study also compared the induction efficiency of exosomes and the indirect coculture system for MSC differentiation. Results showed that NPC exosomes were more effective than an indirect coculture system on MSC differentiation (Figures 5(a) and 5(b)). Rab27a protein is an important molecule in the process of exosome secretion, and knockdown of Rab27a can effectively inhibit exosome secretion [34]. To investigate the role of NPC exosomes in an indirect coculture system, siRNA transfection was performed to knock down Rab27a of NPCs, resulting in the inhibition of NPC exosome secretion and MSC differentiation (Figures 5(d)–5(f)). These results indicated that NPC exosomes mediated the differentiation of MSCs into NP-like cells in an indirect coculture system. It also explained to some extent that the exosome induction efficiency was higher than that of indirect coculture system. In an indirect coculture system, the concentration of exosomes released by NPCs is much lower than that of direct addition of exosomes (50 *μ*g/ml); the release and spread of exosomes also take time, which limits the induction efficiency of an indirect coculture system.

Although exosomes show lots of potentials in stem cell therapy for IDD, there are still some limits in further application. For example, it is difficult to obtain high-purity exosomes efficiently with low cost. Moreover, the contents of the exosomes are related to cell type and status, which may affects the induction efficiency. It is essential to investigate the functional components of exosomes. Further experiments are expected to detect the safety of exosomes in vivo. In addition to the involvement of bioactive factors, cell culture systems also play an important role in stem cell differentiation. It was reported that a 3D environment was very important for adipose-derived stem cell differentiation toward NP-like cells [35, 36]. 3D culture and induction systems have a similar biological environment as that of tissues in vivo [37]. Also, 3D cell culture systems can incorporate bioactive factors, like TGF-*β*, which makes it more effective than monolayer culture systems. Further studies are expected to combine exosomes with 3D cell culture systems on the differentiation of MSCs into NPCs.

## 5. Conclusion

This study confirmed the induction effect of NPC exosomes on the differentiation of MSCs into NP-like cells, and it is more effective than an indirect coculture system. Inhibition of the Notch1 pathway could promote the exosome-induced differentiation of MSCs into NP-like cells. In addition, we also demonstrated that NPC exosomes played a key role in MSC differentiation in an indirect coculture system. This study provided a novel approach for stem cell therapy of IDD.

## Figures and Tables

**Table 1:** The primer sequence of qRT-PCR.

Gene		Primer sequences
Jagged1	Forward	5′-CGCTGTATCTGTCCACCTGGCTAT-3′
	Reverse	5′-TCACTGGCACGATTGTAGCATTGG-3′
Notch1	Forward	5′-TGGAGACAGGCAACAGTGAGGAA-3′
	Reverse	5′-CTTGGCAGCATCTGAACGAGAGTAT-3′
Notch2	Forward	5′-TACCACAACGGCACAGGCTACT-3′
	Reverse	5′-TTCTGACAGCGGTTCTTCTCACAAG-3′
Notch3	Forward	5′-CTGACTGTCTGTTCCTGTCCTCCA-3′
	Reverse	5′-CACCAGCACATTCATCCACATCCT-3′
Notch4	Forward	5′-CTGAAGCGGATGAATGTCGGAGTG-3′
	Reverse	5′-AGCAGGAACACAGCCAGGACTT-3′
Hes1	Forward	5′-CGGACAAACCAAAGACAGCCTCT-3′
	Reverse	5′-TGCCTTCTCCAGCTTGGAATGC-3′
Hey1	Forward	5′-CCGACGAGACCGAATCAATAACAGT-3′
	Reverse	5′-TTCAGCCAGGCATTCCCGAAAC-3′
Collagen II	Forward	5′-CGCTCAAGTCGCTGAACAACCA-3′
	Reverse	5′-ACCAGTTCTTCCGAGGCACAGT-3′
Aggrecan	Forward	5′-TGGCCTGCCTGACTTTAGTG-3′
	Reverse	5′-CCTGAACCACTGACGCTGAT-3′
Sox9	Forward	5′-ACCATCACGCGCTCGCAGT-3′
	Reverse	5′-TGCGCTGGGTTCATGTAGGT-3′
GAPDH	Forward	5′-AGTGCCAGCCTCGTCTCATAGA-3′
	Reverse	5′-GCCTTGACTGTGCCGTTGAACT-3′

Sox9: SRY-related high-mobility group box gene 9; Hes1: hairy and enhancer of split-1; Hey1: Hes-related family BHLH transcription factor with YRPW motif 1.

## Data Availability

The data used to support the findings of this study are available from the corresponding author upon request.

## Supplementary Materials

Supplementary Materials Supplementary Fig. 1: the observation of stained exosomes under the fluorescence microscope. (d) Experimental group: 20 *μ*g exosomes in 100 *μ*l PBS was incubated with CM-Dil in the dark for 30 min. (b) Control group: 100 *μ*l PBS was incubated with CM-Dil in the dark for 30 min. After the incubation, two groups were ultracentrifuged at 120,000 g for 70 min to remove nonbinding dye and then resuspended in PBS. Centrifugation was repeated again. The pellets were resuspended in PBS and then incubated with MSCs (a, c) for 24 h. After the incubation, the fluorescence images were collected by using a fluorescence microscope. Red fluorescence spots were observed in picture (d). On the contrary, there was no fluorescence signal in picture (b). It proved that what we seen in the red channel are stained exosomes but not precipitates of the dye. Supplementary Fig. 2: protein expression of NPC markers (KRT19 and CD24) in exosome-treated MSCs in 7, 14, and 21 days. The expression of KRT19 and CD24 was significantly upregulated in MSCs with the induction of NPC exosomes. Supplementary Fig. 3: morphological comparison of undifferentiated MSCs and differentiated MSCs. (a) The morphology of undifferentiated MSCs. (b) The morphology of MSCs after 21 days of induction of exosomes. Compared with undifferentiated MSCs, the morphology of differentiated MSCs did not change significantly except that they were bigger in size. The induction effects of exosomes on the differentiation of MSCs into NPCs were mainly reflected in the upregulated expression of NP markers in MSCs, rather than morphology.
