# Supplementary material for: Inhibition of the Notch1 Pathway Promotes the Effects of Nucleus Pulposus Cell-Derived Exosomes on the Differentiation of Mesenchymal Stem Cells into Nucleus Pulposus-Like Cells in Rats
Source: Stem Cells Int. 2019 May 6;2019:8404168. doi: 10.1155/2019/8404168 (PMC6526523; doi:10.1155/2019/8404168)
Supplement: Supplementary Materials — Supplementary Fig. 1: the observation of stained exosomes under the fluorescence microscope. (d) Experimental group: 20 μg exosomes in 100 μl PBS was incubated with CM-Dil in the dark for 30 min. (b) Control group: 100 μl PBS was incubated with CM-Dil in the dark for 30 min. After the incubation, two groups were ultracentrifuged at 120,000 g for 70 min to remove nonbinding dye and then resuspended in PBS. Centrifugation was repeated again. The pellets were resuspended in PBS and then incubated with MSCs (a, c) for 24 h. After the incubation, the fluorescence images were collected by using a fluorescence microscope. Red fluorescence spots were observed in picture (d). On the contrary, there was no fluorescence signal in picture (b). It proved that what we seen in the red channel are stained exosomes but not precipitates of the dye. Supplementary Fig. 2: protein expression of NPC markers (KRT19 and CD24) in exosome-treated MSCs in 7, 14, and 21 days. The expression of KRT19 and CD24 was significantly upregulated in MSCs with the induction of NPC exosomes. Supplementary Fig. 3: morphological comparison of undifferentiated MSCs and differentiated MSCs. (a) The morphology of undifferentiated MSCs. (b) The morphology of MSCs after 21 days of induction of exosomes. Compared with undifferentiated MSCs, the morphology of differentiated MSCs did not change significantly except that they were bigger in size. The induction effects of exosomes on the differentiation of MSCs into NPCs were mainly reflected in the upregulated expression of NP markers in MSCs, rather than morphology. [file 8404168.f1.doc]

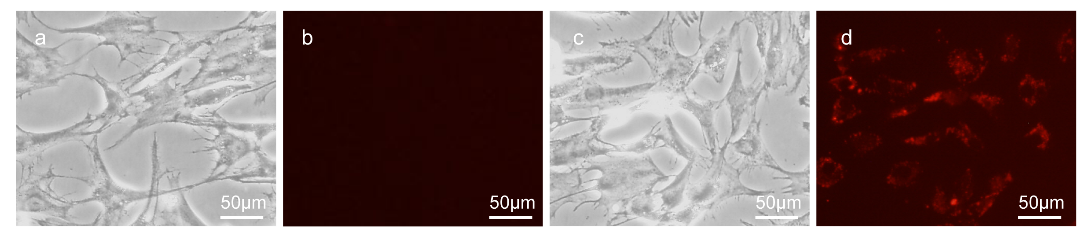


**Supplementary Fig.1:** The observation of stained exosomes under the fluorescence microscope. (d) Experimental group: 20 µg exosomes in 100µl PBS was incubated with CM-Dil in the dark for 30 min; (b) Control group: 100µl PBS was incubated with CM-Dil in the dark for 30 min. After the incubation, two groups were ultra-centrifuged at 120 000 g for 70 min to remove non-binding dye, then resuspended in PBS. Repeat the centrifugation again. The pellets were resuspended in PBS and then incubated with MSCs (a)(c) for 24h. After the incubation, the fluorescence images were collected by fluorescence microscope. Red fluorescence spots were observed in picture d. On the contrary, there was no fluorescence signal in picture b. It proved that what we seen in the red channel are stained exosomes but not precipitates of the dye.


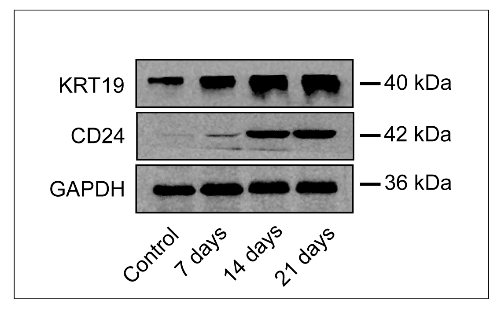


**Supplementary Fig.2:** Protein expression of NPC markers (KRT19, CD24) in exosomes-treated MSCs in 7, 14, 21 days. The expression of KRT19 and CD24 were significantly upregulated in MSCs with the induction of NPC exosomes.


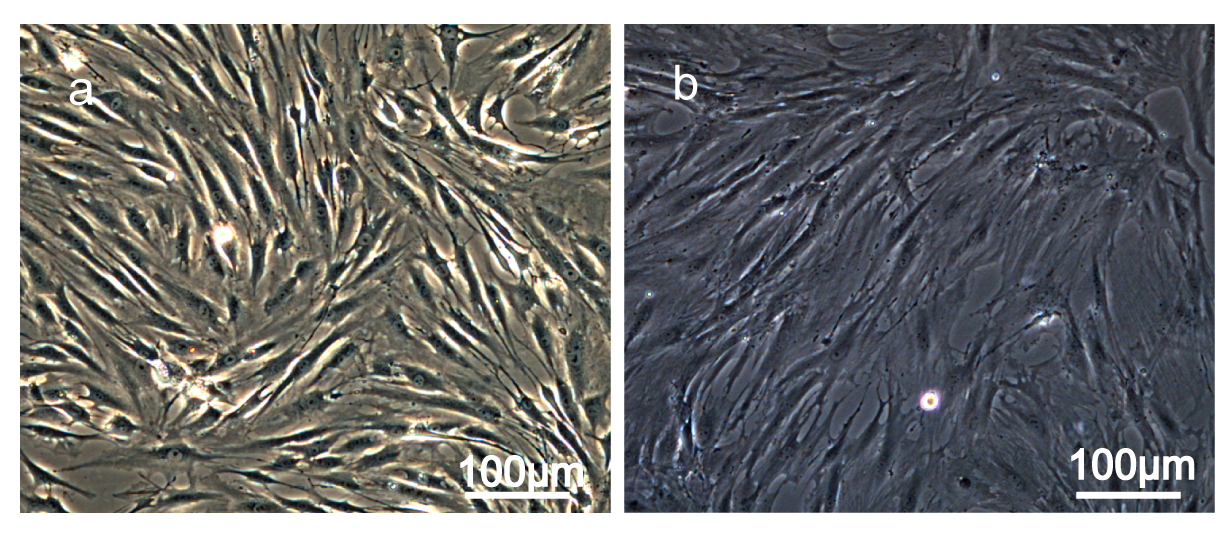


**Supplementary Fig3:** Morphological comparison of undifferentiated MSCs and differentiated MSCs. (a): The morphology of undifferentiated MSCs. (b): The morphology of MSCs after 21 days’ induction of exosomes. compared with undifferentiated MSCs, the morphology of differentiated MSCs did not change significantly except bigger in size. The inducing effects of exosomes on the differentiation of MSCs into NPCs were mainly reflected in the up-regulated expression of NPmarkers in MSCs, rather than morphology.
